# Supplementary material for: Fabricating supramolecular pre-emergence herbicide CPAM-BPyHs for farming herbicide-resistant rice
Source: Nat Commun. 2025 May 10;16:4347. doi: 10.1038/s41467-025-59582-9 (PMC12065884; doi:10.1038/s41467-025-59582-9)
Supplement: Supplementary file 1 — Supplementary Information [file 41467_2025_59582_MOESM1_ESM.pdf]

Fabricating supramolecular pre-emergence herbicide CPAM-BPyHs for farming herbicide-resistant rice

Chen *et al*.

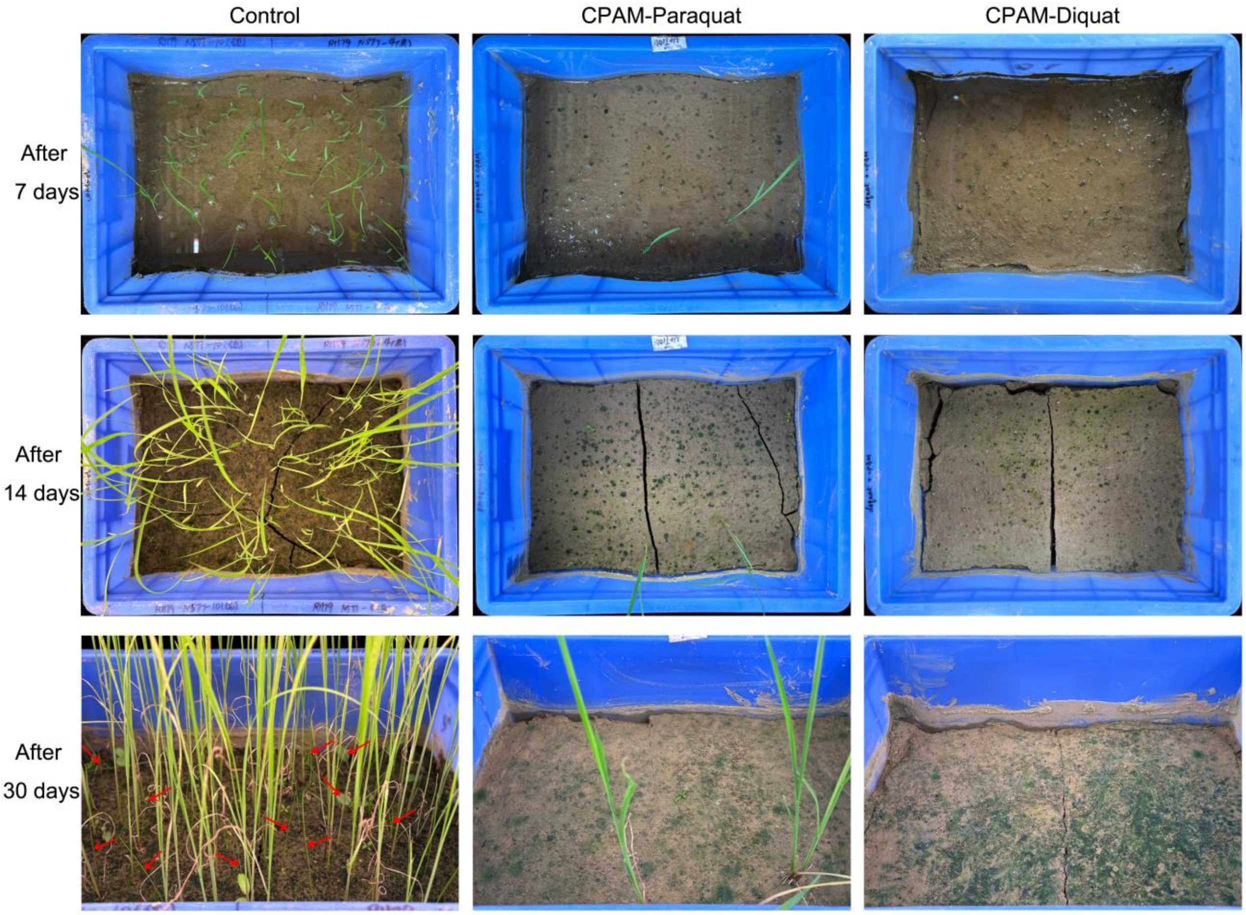

Supplementary Fig. 1. Weed growth at different times after soil treated with CPAM-BPyHs. Control refers to the treatment with only water on the 1st day. CPAM-Paraquat refers to the treatment with 1‰ CPAM-5 mM paraquat at the 1st day. CPAM-Diquat refers to the treatment with 1‰ CPAM-5 mM diquat at the 1st day. The red arrow sites are new weeds.

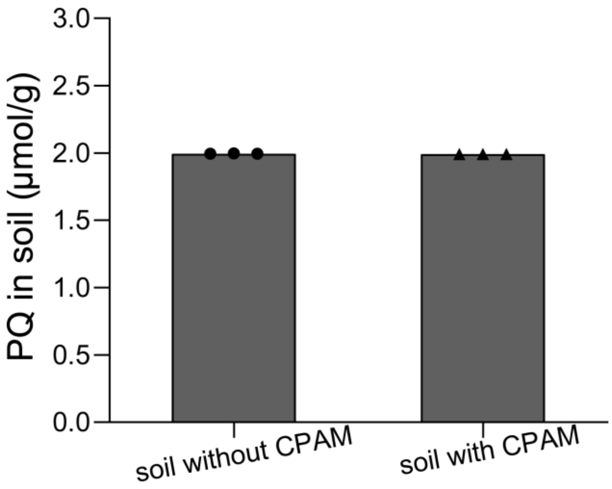

Supplementary Fig. 2. The content of adsorbed paraquat in soil with or without CPAM. Difference was analyzed using the two-tailed unpaired *t*-test. Data represent mean ± SD. n = 3 independent experiments. Source data are provided as a Source Data file.

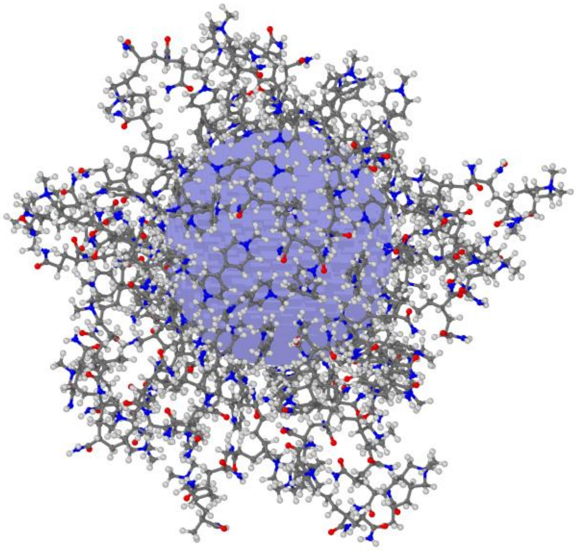

Supplementary Fig. 3. Snapshot of paraquat and CPAM absorbed on soil particle after 6×10^6 timesteps MD simulations in soil-CPAM-paraquat system.

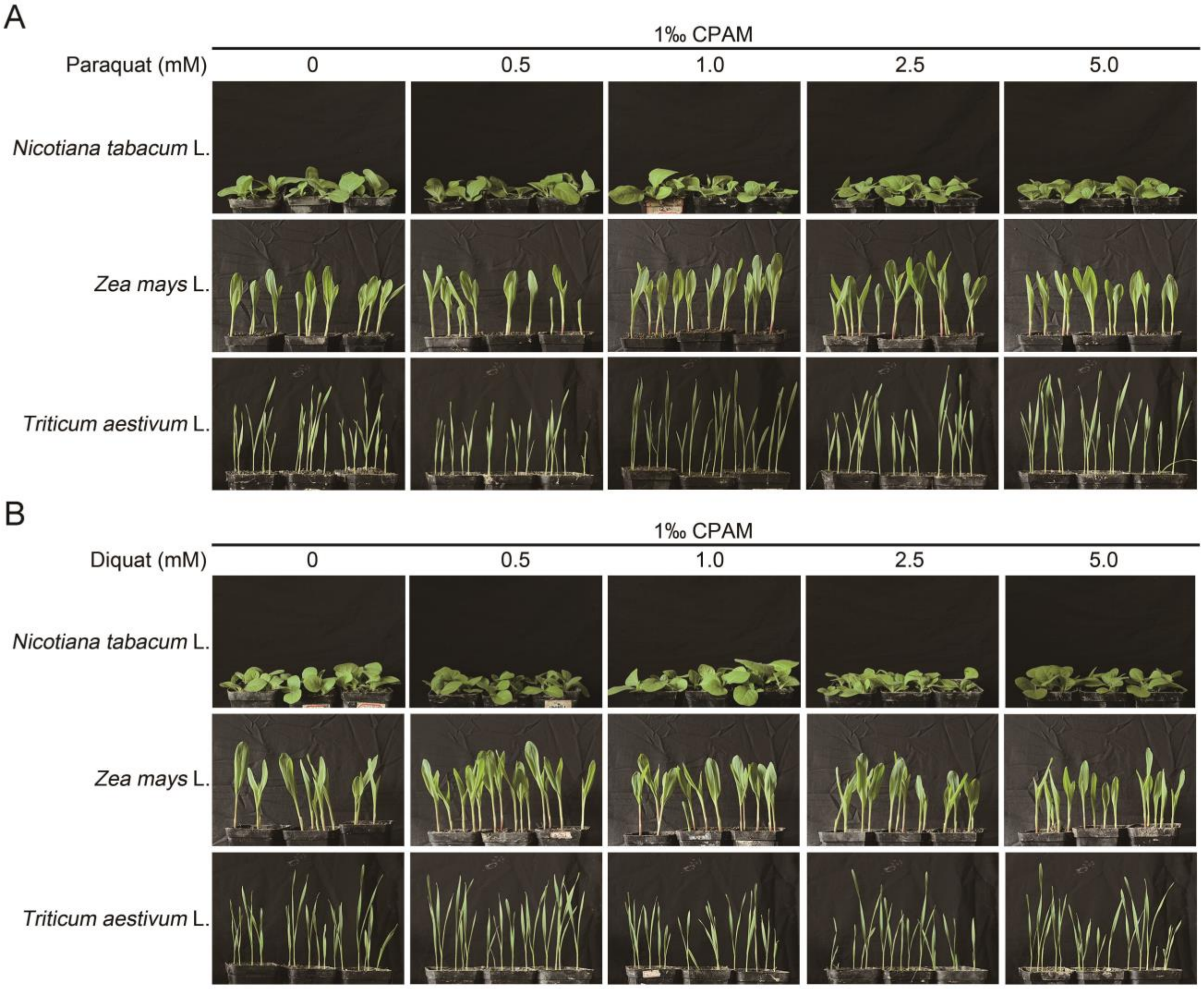

Supplementary Fig. 4. The effects of (A) CPAM-paraquat or (B) CPAM-diquat on different succeeding crops. Tobacco (*Nicotiana tabacum* L.), corn (*Zea mays* L.) and wheat (*Triticum aestivum* L.) were sown in the soil, which was treated with CPAM-BPyHs 60 days before sowing the seeds.

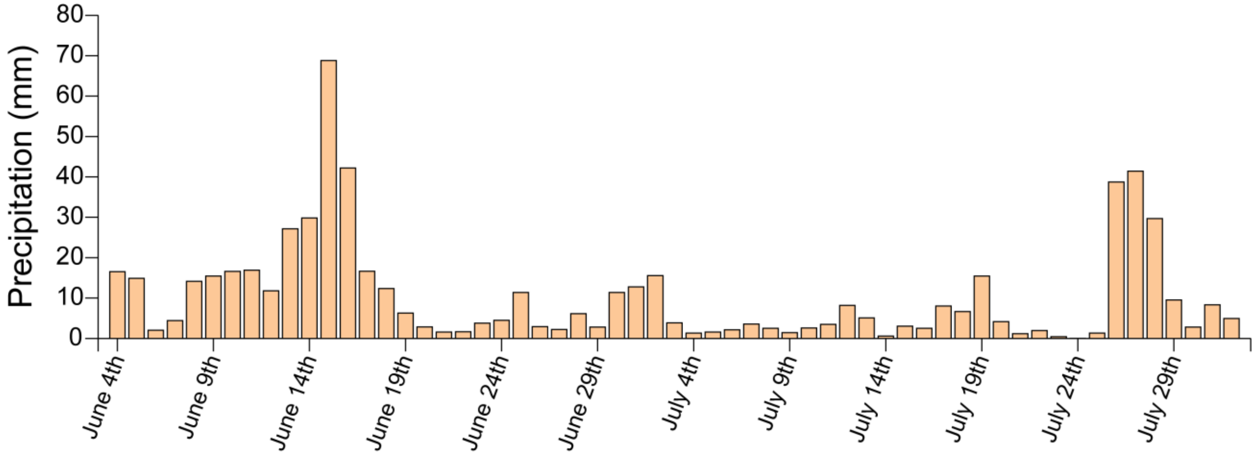

Supplementary Fig. 5. Rainfall during the experimental period. The rainfall data were from the Xihe Energy Weather Big Data Platform (www.xihe-energy.com, accessed on September 25th, 2024). Source data are provided as a Source Data file.

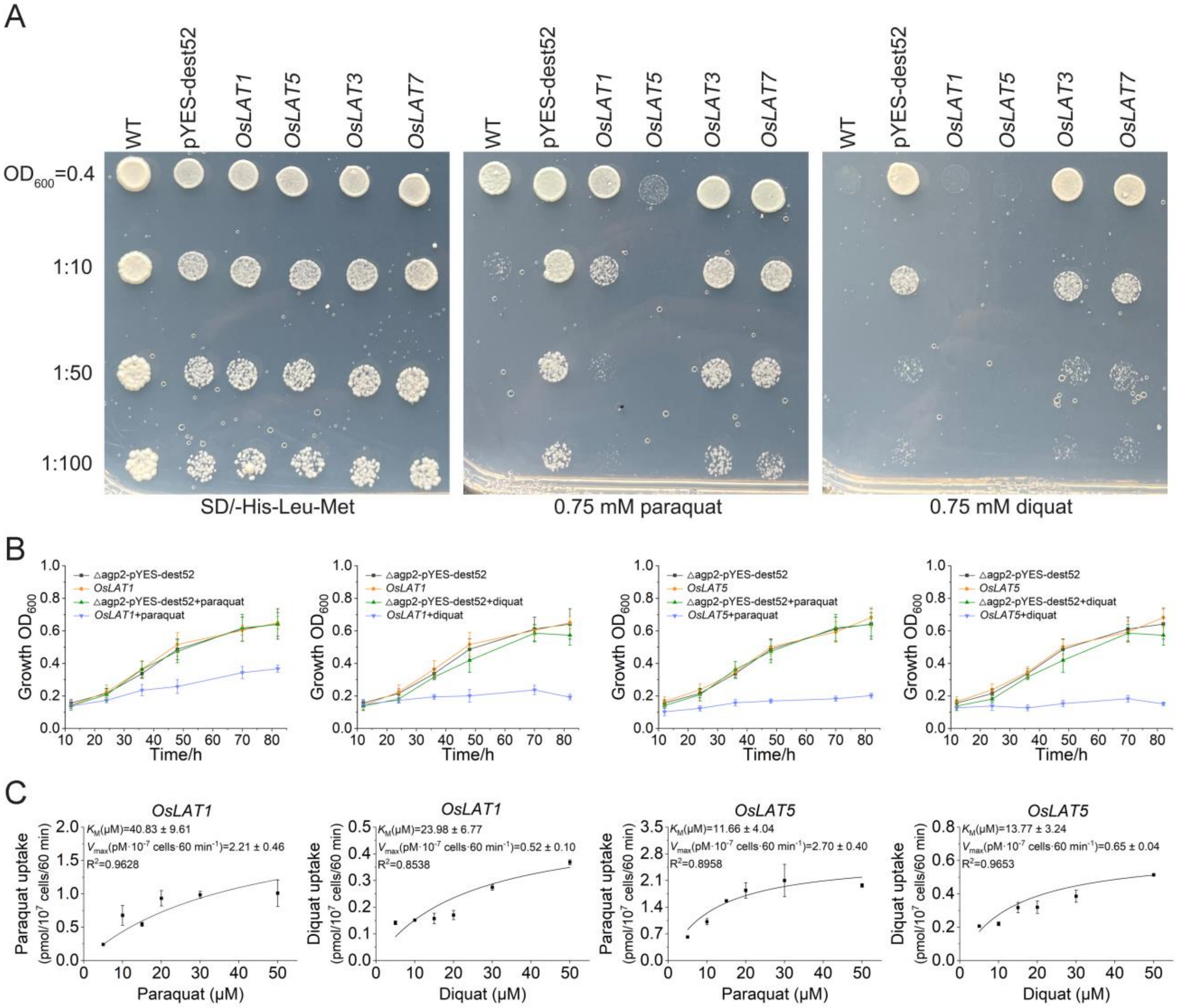

Supplementary Fig. 6. The function of OsLATs as BPyHs transporter in yeast heterologous system. (**A**) Growth of yeast mutant Δ*agp2* transformants carrying four *LATs* (1, 3, 5 and 7) genes. (**B**) Growth curves plotted from OD_{600} values of liquid cultures. Yeast cells were grown in liquid medium containing 0.75 mM paraquat or diquat. (**C**) Dose-dependent and kinetic analysis of BPyHs uptaken by *OsLAT1* and *OsLAT5*. Michaelis–Menten curves for BPyHs uptake were obtained by subtracting the uptake in vector control expressing cells from that in Δ*agp2-OsLAT1* or *OsLAT5* cells. Data represent mean ± SD. n = 2 yeast cell samples. Source data are provided as a Source Data file.

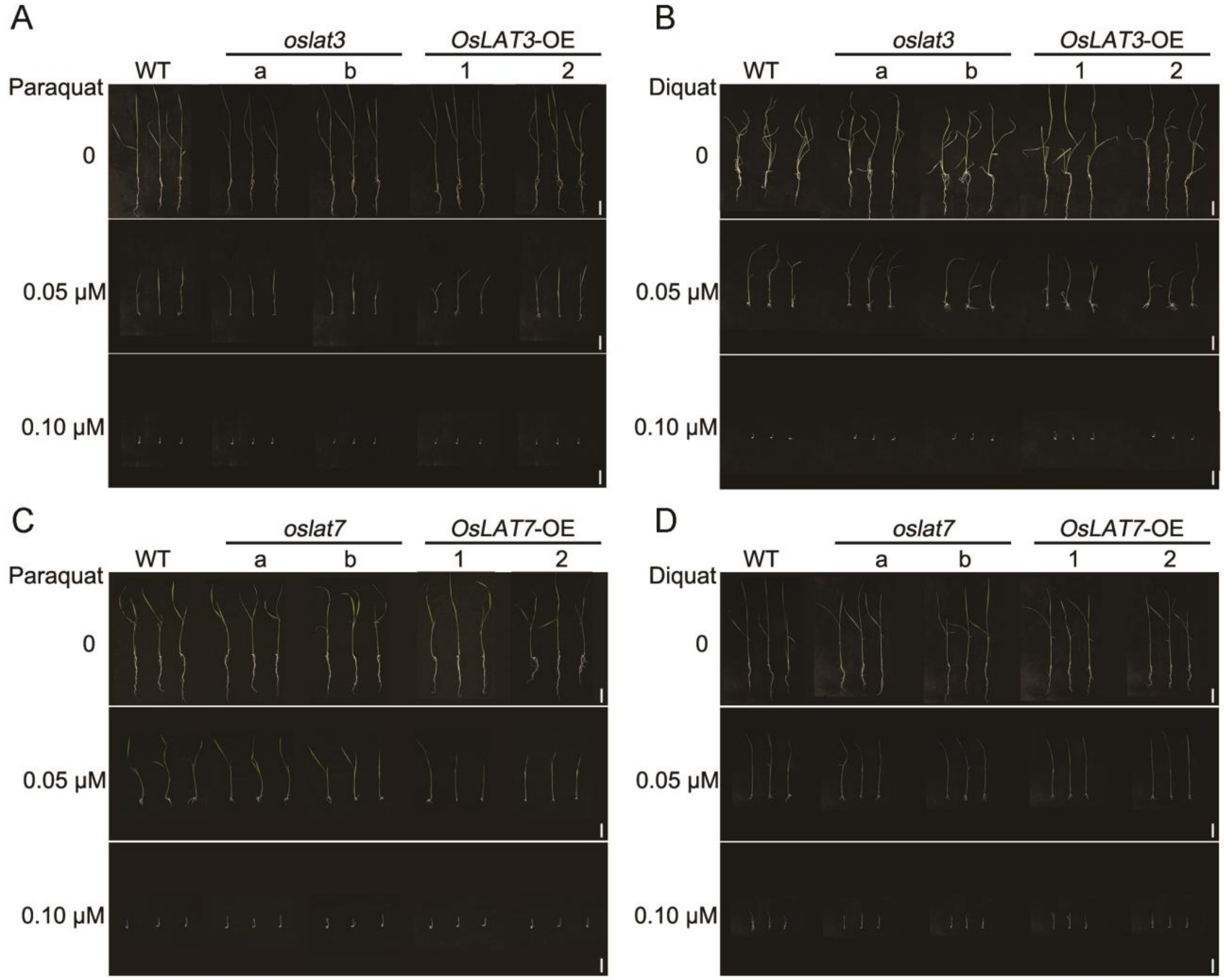

Supplementary Fig. 7. Rice seed germination and seedling growth in BPyHs by *OsLAT3* and *OsLAT7*. Growth of germinated rice seedlings mediated by *OsLAT3* with (**A**) paraquat or (**B**) diquat on MS medium. Bar = 4 cm. Growth of germinated rice seedlings mediated by *OsLAT7* with (**C**) paraquat or (**D**) diquat on MS medium. Bar = 4 cm.

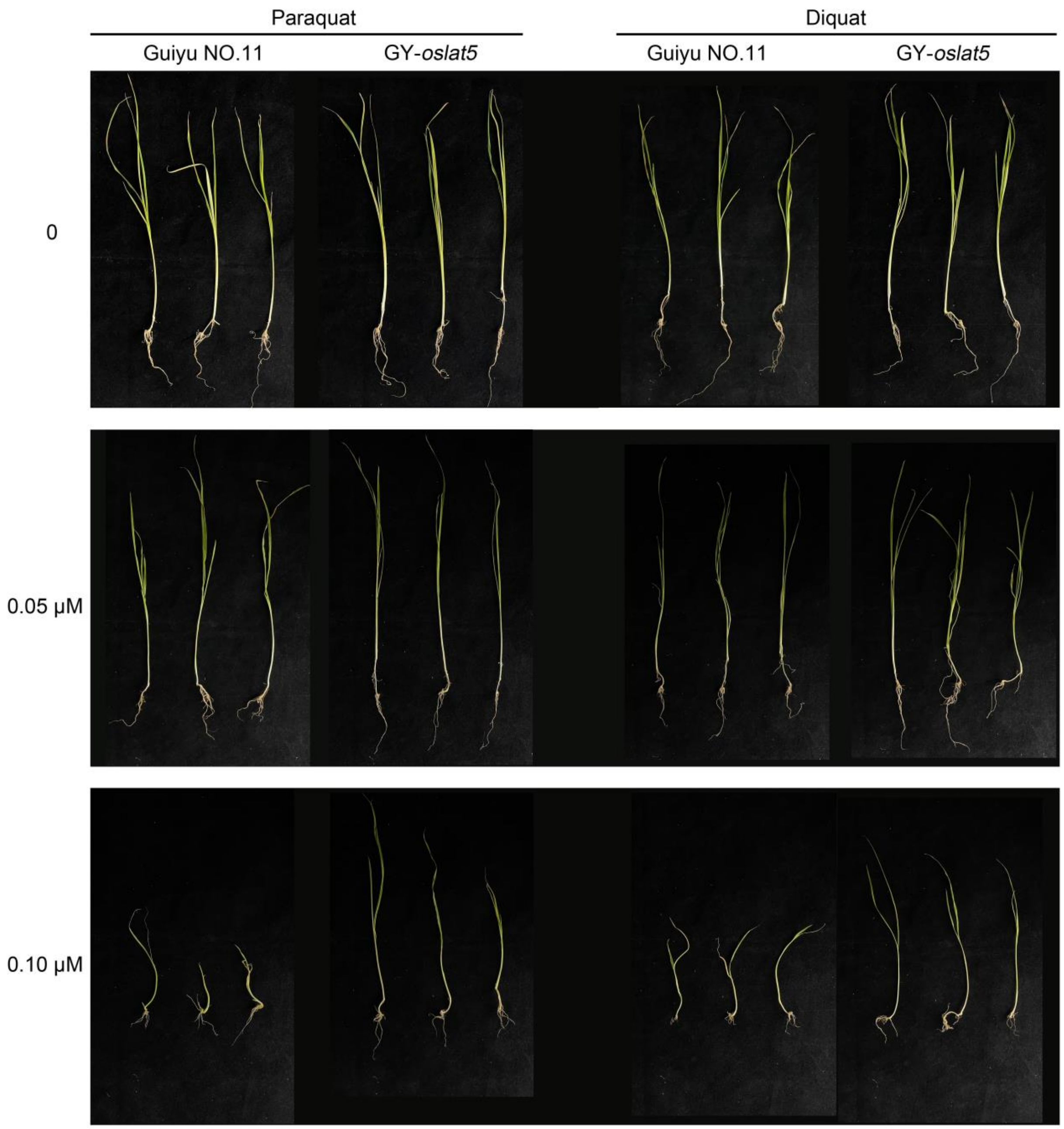

Supplementary Fig. 8. The GY-*oslat5* mutant (Guiyu NO.11 background) phenotype on paraquat or diquat medium.

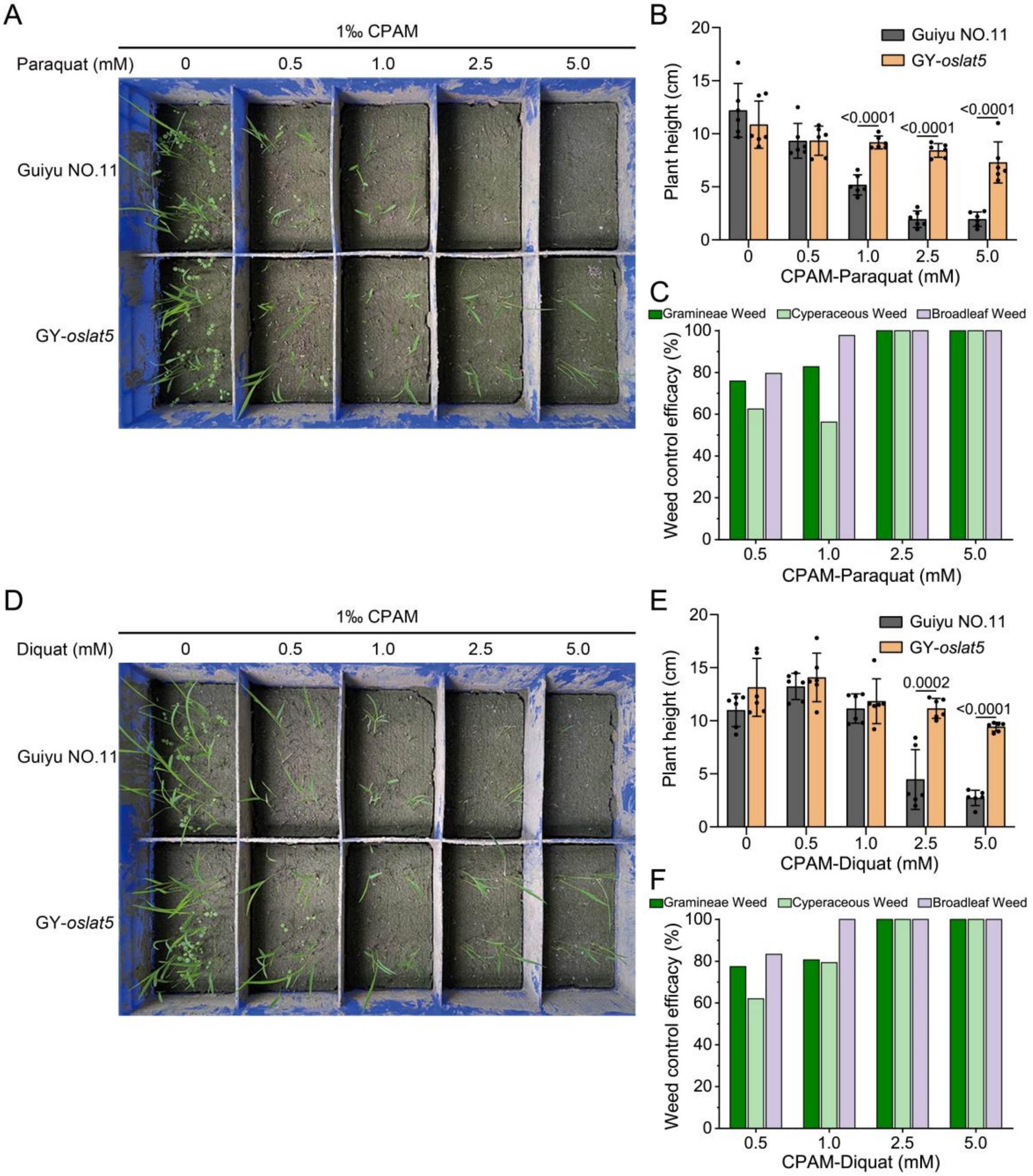

Supplementary Fig. 9. Indoor crop-weed competition of Guiyu NO.11, GY-*oslat5* and weeds in soil treated with CPAM-paraquat or CPAM-diquat. Growth of Guiyu NO.11 and GY-*oslat5* in soil treated with (**A**) CPAM-paraquat or (**D**) CPAM-diquat. Plant height of Guiyu NO.11 and GY-*oslat5* in soil treated with (**B**) CPAM-paraquat or (**E**) CPAM-diquat. Difference was analyzed using the multiple *t*-test. Data represent mean ± SD. n = 6 rice plants. Weed control efficacy in soil treated with (**C**) CPAM-paraquat or (**F**) CPAM-diquat. Source data are provided as a Source Data file.

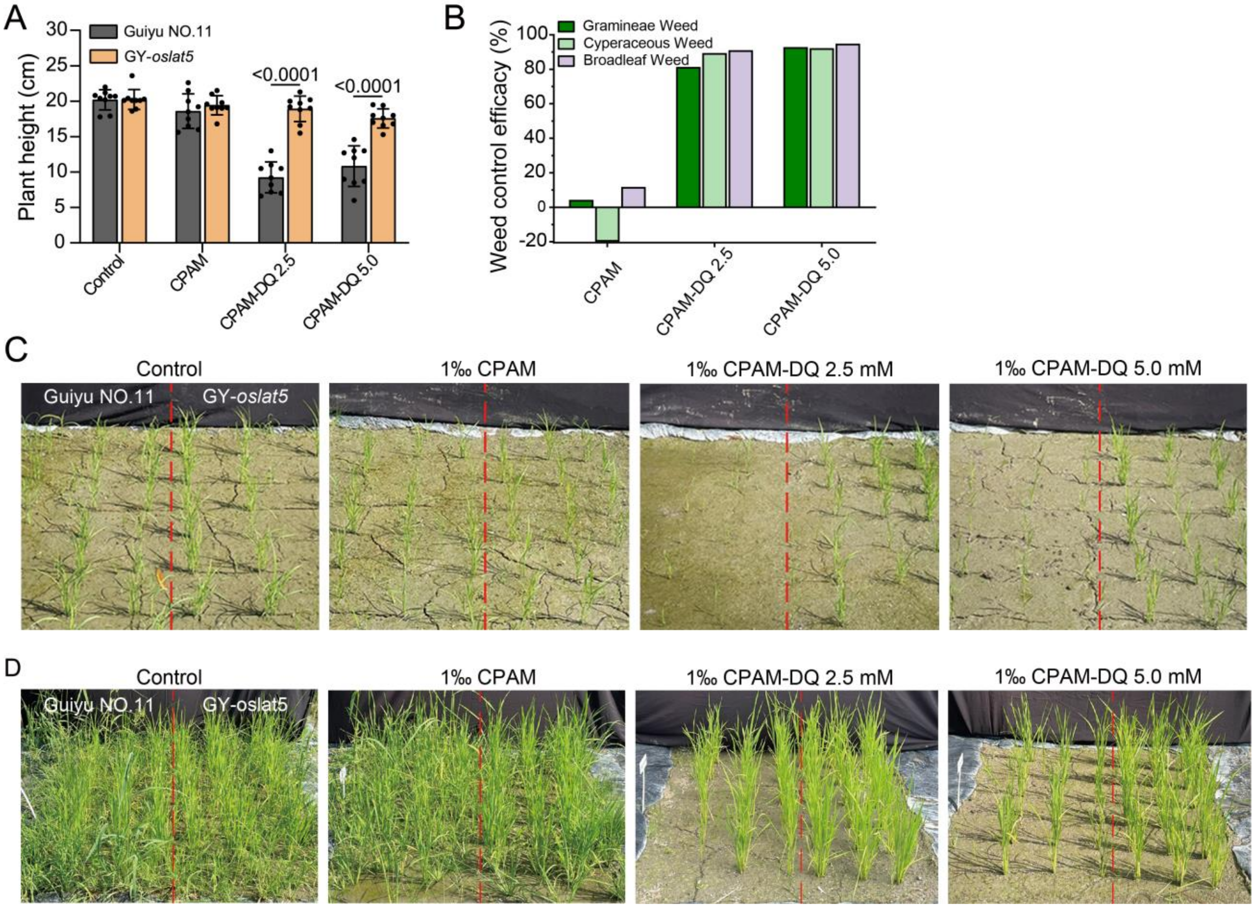

Supplementary Fig. 10. Field crop-weed competition of Guiyu NO.11, GY-*oslat5* and weeds in soil treated with CPAM-diquat. (**A**) Plant height of Guiyu NO.11 and GY-*oslat5* after 10 days. Difference was analyzed using the multiple *t*-test. Data represent mean ± SD. n = 9 rice plants. (**B**) Weed control efficacy after 10 days. (**C**) Field performance after 10 days. (**D**) Field performance after 30 days. Control refers to the treatment with only water. DQ refers to diquat. Source data are provided as a Source Data file.

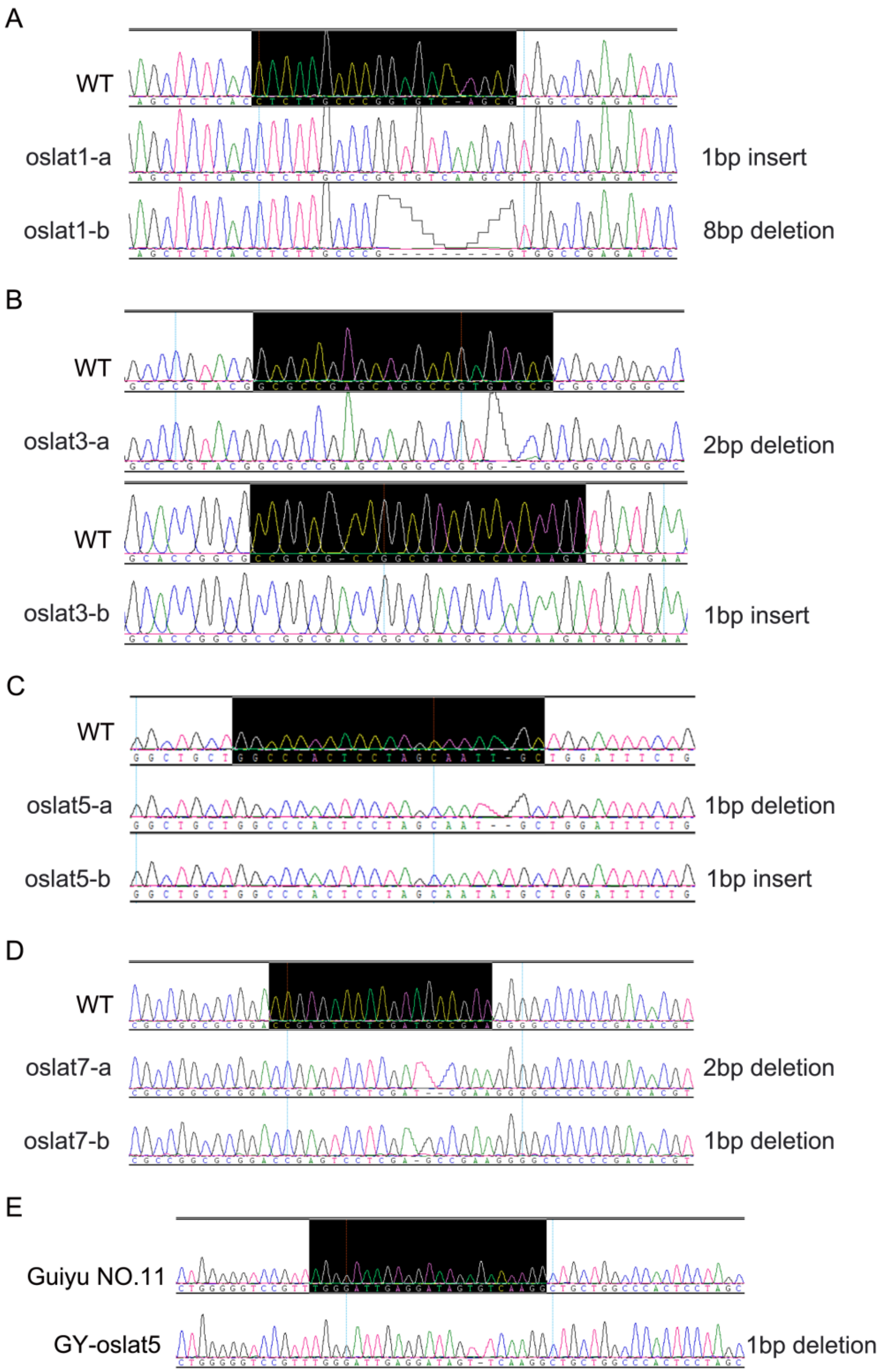

Supplementary Fig. 11. *OsLAT* knockout mutants in rice. (**A**) *oslat1*, (**B**) *oslat3*, (**C**) *oslat5* and (**D**) *oslat7* knockout mutants generated in Zhonghua11 background. (**E**) *oslat5* knockout mutant generated in Guiyu NO.11 background.

Supplementary Table 1. Primers sequences used in this study.

Name	**Primer sequence** (Bold font indicates vector sequence)	Note
pENTR-*OsLAT1*	P1: **ACCAATTCAGTCGACTGGATCC**ATGGCGGACACCGGCGGAC	Vector: pENTR 2B Dual
	P2: **TACAAGAAAGCTGGGTCTAGATATC**CTACGGAACCAACGGCTCG	
pENTR-*OsLAT5*	P3: **ACCAATTCAGTCGACTGGATCC**ATGGAGGATTGTGTTGGTAT	
	P4: **TACAAGAAAGCTGGGTCTAGATATC**TCAGCACACAAGTGGGATT	
pENTR-*OsLAT7*	P5: **ACCAATTCAGTCGACTGGATCC**ATGACCGGAGCCTGCGAGG	
	P6: **TACAAGAAAGCTGGGTCTAGATATC**CTAATAATTCTCCTTGCTGACAC	
pENTR-*OsLAT3*	P7: **ACCAATTCAGTCGACTGGATCC**ATGAGTGAGCTCACCATGGAC	
	P8: **TACAAGAAAGCTGGGTCTAGATATC**CTACGTACCTGCTGCGTCAG	
35S-*OsLAT1*	P9: **ACCCGGGGATCCTCTAGAGTCGA**ATGGCGGACACCGGCGGAC	Vector: pCAMB1300-35s
	P10: **ATGATACGAACGAAAGCTCTGCA**CTACGGAACCAACGGCTCG	
35S-*OsLAT5*	P11: **ACCCGGGGATCCTCTAGAGTCGA**ATGGAGGATTGTGTTGGTAT	
	P12: **ATGATACGAACGAAAGCTCTGCA**TCAGCACACAAGTGGGATT	
35S-*OsLAT7*	P13: **ACCCGGGGATCCTCTAGAGTCGA**ATGACCGGAGCCTGCGAGG	
	P14: **ATGATACGAACGAAAGCTCTGCA**CTAATAATTCTCCTTGCTGACAC	
35S-*OsLAT3*	P15: **ACCCGGGGATCCTCTAGAGTCGA**ATGAGTGAGCTCACCATGGAC	
	P16: **ATGATACGAACGAAAGCTCTGCA**CTACGTACCTGCTGCGTCAG	
Actin (UBQ2)	P17: TGCTATGTACGTCGCCATCCAG	For identifying OE rice lines
	P18: AATGAGTAACCACGCTCCGTCA	
qRT-PCR-*OsLAT1*	P19: ATCGGCTTCTTGGTCCTCC	
	P20: CCACTTCATCCACCCTTGC	
qRT-PCR-*OsLAT5*	P21: CGCCTCCCGACCTTACAA	
	P22: GCACGAACCCAACCAGCA	
qRT-PCR-*OsLAT7*	P23: GCCTGACAGCCTCCGTG	
	P24: CTGACACTGACGCCTTCTCC	
qRT-PCR-*OsLAT3*	P25: TGATGAACAGCAAGGGCACC	
	P26: GGAGTAGCGTGAGCTTGGTCTG	
Crispr-*OsLAT1*	P27: GTAGCGATGCGACGAGAGAG	For identifying rice mutants
	P28: AAATGTGCTGCGCGCTTTAT	
Crispr-*OsLAT5*	P29: GTGTTGCATGGTGAAACAAGGA	
	P20: CAAGGGCTGAAGAGACCCAG	
Crispr-GY-*oslat5*	P31: GCTAACTCAGCCTGTCTTCC	
	P32: AAGTAAGTGCAACTGTGAGG	
Crispr-*OsLAT7*	P33: CTCGTCGAACACCTTGTCGC,	
	P34: TTGGAGAACCCGACGAGGAA	
Crispr-*OsLAT3-a*	P35: AGATGATGAACAGCAAGGGC	
	P36: GACGAAGCCGCCGTTCCCCG	
Crispr-*OsLAT3-b*	P37: AACTGAACCAGAGGCCATCGC	
	P38: ACAATGCTTAGGCCGCCCAA	
