# Supplementary material for: Fabricating supramolecular pre-emergence herbicide CPAM-BPyHs for farming herbicide-resistant rice
Source: Nat Commun. 2025 May 10;16:4347. doi: 10.1038/s41467-025-59582-9 (PMC12065884; doi:10.1038/s41467-025-59582-9)
Supplement: Supplementary file 3 — Reporting Summary [file 41467_2025_59582_MOESM3_ESM.pdf]

Corresponding author(s): Lingda Zeng, Fei Lin, Hanhong Xu

Last updated by author(s): Apr 14, 2025

Reporting Summary

Nature Portfolio wishes to improve the reproducibility of the work that we publish. This form provides structure for consistency and transparency in reporting. For further information on Nature Portfolio policies, see our Editorial Policies and the Editorial Policy Checklist.

Statistics

For all statistical analyses, confirm that the following items are present in the figure legend, table legend, main text, or Methods section.

n/a	Confirmed	
☐	☒	The exact sample size (*n*) for each experimental group/condition, given as a discrete number and unit of measurement
☐	☒	A statement on whether measurements were taken from distinct samples or whether the same sample was measured repeatedly
☐	☒	The statistical test(s) used AND whether they are one- or two-sided<br>*Only common tests should be described solely by name; describe more complex techniques in the Methods section.*
☒	☐	A description of all covariates tested
☒	☐	A description of any assumptions or corrections, such as tests of normality and adjustment for multiple comparisons
☐	☒	A full description of the statistical parameters including central tendency (e.g. means) or other basic estimates (e.g. regression coefficient) AND variation (e.g. standard deviation) or associated estimates of uncertainty (e.g. confidence intervals)
☐	☒	For null hypothesis testing, the test statistic (e.g. *F*, *t*, *r*) with confidence intervals, effect sizes, degrees of freedom and *P* value noted<br>*Give P values as exact values whenever suitable.*
☒	☐	For Bayesian analysis, information on the choice of priors and Markov chain Monte Carlo settings
☒	☐	For hierarchical and complex designs, identification of the appropriate level for tests and full reporting of outcomes
☒	☐	Estimates of effect sizes (e.g. Cohen's *d*, Pearson's *r*), indicating how they were calculated

Our web collection on statistics for biologists contains articles on many of the points above.

Software and code

Policy information about availability of computer code

Data collection	1. Measurement of OD600 in yeast assay: Bio-rad, iMarkTM Microplate Reader;<br>2. Measurement of bipyridyl herbicide: UPLC-MS/MS, chromatographic separation was carried out on an ACQUITY UPLC® H-Class system (Waters) with a CORTECS UPLC HILIC column (2.1 × 100 mm, 1.7 µm; Waters), MS analysis was carried out on a Waters Xevo TQD triple-quadrupole mass spectrometer with an ESI source.<br>3. qRT-PCR: Bio-rad, CFX96;<br>4. Measurement of specific surface area: JWGB INSTRUMENTS, TB440A;<br>5. Measurement of zeta potential: Zetasizer, Nano ZSE;<br>6. Molecular dynamics (MD) simulations: all the MD simulations were implemented using the Large-scale Atomic/Molecular Massively Parallel Simulator (LAMMPS) package.<br>7.The rainfall data were from the Xihe Energy Weather Big Data Platform (www.xihe-energy.com, accessed on 25th September 2024).
Data analysis	For UPLC-MS/MS measurements, data were analyzed using Waters MassLynx version 4.1. Gene sequencing results were viewed by SnapGene3.2. Data were visualized using Microsoft Excel 2019, GraphPad Prism 8 and Origin 2021. The significant differences in values obtained were analyzed using GraphPad Prism 8.

For manuscripts utilizing custom algorithms or software that are central to the research but not yet described in published literature, software must be made available to editors and reviewers. We strongly encourage code deposition in a community repository (e.g. GitHub). See the Nature Portfolio guidelines for submitting code & software for further information.

Data

Policy information about availability of data

All manuscripts must include a data availability statement. This statement should provide the following information, where applicable:

- Accession codes, unique identifiers, or web links for publicly available datasets
- A description of any restrictions on data availability
- For clinical datasets or third party data, please ensure that the statement adheres to our policy

Source data are provided with this paper.

Research involving human participants, their data, or biological material

Policy information about studies with human participants or human data. See also policy information about sex, gender (identity/presentation), and sexual orientation and race, ethnicity and racism.

Reporting on sex and gender	This study did not involve human participants, their data or biological material.
Reporting on race, ethnicity, or other socially relevant groupings	This study did not involve human participants, their data or biological material.
Population characteristics	This study did not involve human participants, their data or biological material.
Recruitment	This study did not involve human participants, their data or biological material.
Ethics oversight	This study did not involve human participants, their data or biological material.

Note that full information on the approval of the study protocol must also be provided in the manuscript.

Field-specific reporting

Please select the one below that is the best fit for your research. If you are not sure, read the appropriate sections before making your selection.

- [x] Life sciences
- [ ] Behavioural & social sciences
- [ ] Ecological, evolutionary & environmental sciences

For a reference copy of the document with all sections, see nature.com/documents/nr-reporting-summary-flat.pdf

Life sciences study design

All studies must disclose on these points even when the disclosure is negative.

Sample size	The sample size and the results of statistical analyses were described in the figure legends. More specifically, in herbicide resistance evaluation of different rice lines in MS medium, wild type and genotype line were planted in the same tissue culture bottle, and there were 24 seedlings in each bottle, then 3 seedlings of different lines were randomly selected to record and photograph. In evaluation of herbicidal activities in indoor test, 6 rice seedlings of each treatment were recorded, all the weeds of each treatment were counted. In evaluation of herbicidal activities in field trials, 9 rice seedlings of each treatment were recorded, weeds in 5 sites (21.4 cm*21.4 cm) of each treatment were counted. In large-area field trial, 6 sites (1 m*1 m) were randomly selected in each field to survey the number of weeds.
Data exclusions	No data were excluded from the analyses.
Replication	The main experiments in the article, including herbicide resistance evaluation of different rice lines in MS medium, evaluation of herbicidal activities in indoor test and yeast growth in solid medium et al., were done in triplicates and were all reproducible.
Randomization	Samples were randomly collected.
Blinding	In studying adsorption behavior of herbicides in soil, measurement of specific surface area and zeta potential, safety evaluation of soil treating with CPAM-herbicide on neighboring crops or neighboring crops, evaluation of herbicidal activities in field trials, herbicide resistance evaluation of different rice lines in MS medium, the researchers were blinded as to which samples were being analyzed. In comparison of soil treating with CPAM-diquat and foliar spraying with diquat, the treatment methods and application time of different groups were different, so the blind trial could not be conducted. Given that CPAM-herbicide had already demonstrated good herbicidal efficacy in field trials, the large-scale field trial focused on showing the effect of CPAM-diquat. Therefore, a blinded experimental design was not required.

Reporting for specific materials, systems and methods

We require information from authors about some types of materials, experimental systems and methods used in many studies. Here, indicate whether each material, system or method listed is relevant to your study. If you are not sure if a list item applies to your research, read the appropriate section before selecting a response.

Materials & experimental systems

n/a	Involved in the study	
☒	☐	Antibodies
☒	☐	Eukaryotic cell lines
☒	☐	Palaeontology and archaeology
☒	☐	Animals and other organisms
☒	☐	Clinical data
☒	☐	Dual use research of concern
☐	☒	Plants

Methods

n/a	Involved in the study	
☒	☐	ChIP-seq
☒	☐	Flow cytometry
☒	☐	MRI-based neuroimaging

Dual use research of concern

Policy information about dual use research of concern

Hazards

Could the accidental, deliberate or reckless misuse of agents or technologies generated in the work, or the application of information presented in the manuscript, pose a threat to:

No	Yes	
☒	☐	Public health
☒	☐	National security
☒	☐	Crops and/or livestock
☒	☐	Ecosystems
☒	☐	Any other significant area

Experiments of concern

Does the work involve any of these experiments of concern:

No	Yes	
☒	☐	Demonstrate how to render a vaccine ineffective
☒	☐	Confer resistance to therapeutically useful antibiotics or antiviral agents
☒	☐	Enhance the virulence of a pathogen or render a nonpathogen virulent
☒	☐	Increase transmissibility of a pathogen
☒	☐	Alter the host range of a pathogen
☒	☐	Enable evasion of diagnostic/detection modalities
☒	☐	Enable the weaponization of a biological agent or toxin
☒	☐	Any other potentially harmful combination of experiments and agents

Plants

Seed stocks	All the rice genotype materials were constructed from EDGENE Biotechnology (Wuhan) Co., Ltd.
Novel plant genotypes	The rice mutants oslat1, 3, 5, 7 (Zhonghua 11 background) were obtained via CRISPR/Cas9. The rice mutants oslat5 (Guiyu NO.11 background) was obtained via CRISPR/Cas9. The recombinant vector containing target sequence and sg-RNA was recombined with the vector pH-Ubicas9-7 containing Cas9 by LR mix to form a complete recombinant vector containing target sequence -sg-RNA +Cas9, then rice mutants were obtained by introducing recombinant vector into rice callus. For obtaining overexpression rice, the pCAMBIA1300-35S vectors, carrying different OsLAT genes, were transformed into Zhonghua11 with Agrobacterium tumefaciens-mediated transformation.
Authentication	The mutant was identified by sequencing PCR products which were amplified with gene-specific primers (Supplementary Table 1), and also the Cas9 sequence was not detected. The overexpression rice lines were confirmed by 50 mg/L hygromycin resistance and gene expression level analysis.
